# Regorafenib Induces Senescence and Epithelial-Mesenchymal Transition in Colorectal Cancer to Promote Drug Resistance

**DOI:** 10.3390/cells11223663

**Published:** 2022-11-18

**Authors:** Pashalina Kehagias, Nadège Kindt, Mohammad Krayem, Ahmad Najem, Giulia Agostini, Elena Acedo Reina, Giacomo Bregni, Francesco Sclafani, Fabrice Journe, Ahmad Awada, Ghanem E. Ghanem, Alain Hendlisz

**Affiliations:** 1 GUTS Lab., Department of Medical Oncology, Institut Jules Bordet, Université Libre de Bruxelles, Rue Meylemeersch 90, 1070 Anderlecht, Belgium; 2 Laboratory of Clinical and Experimental Oncology, Institut Jules Bordet, Université Libre de Bruxelles (ULB), Rue Meylemeersch 90, 1070 Anderlecht, Belgium; 3 Department of Medical Oncology, Institut Jules Bordet, Université Libre de Bruxelles, Rue Meylemeersch 90, 1070 Anderlecht, Belgium

**Keywords:** regorafenib, CRC, phenotype switch, senescence, EMT

## Abstract

Potential intrinsic resistance mechanisms to regorafenib were explored after short exposure (3 days) on five CRC cell lines (HCT-116, SW1116, LS-1034, SW480, Caco-2). The observation of senescence-like features led to the investigation of a drug-initiated phenotype switch. Following long-term exposure (12 months) of HCT-116 and SW480 cell lines to regorafenib, we developed resistant models to explore acquired resistance. SW480 cells demonstrated senescent-like properties, including a cell arrest in the late G2/prophase cell cycle stage and a statistically significant decrease in the expression of G1 Cyclin-Dependent Kinase inhibitors and key cell cycle regulators. A specific senescence-associated secretome was also observed. In contrast, HCT-116 treated cells presented early senescent features and developed acquired resistance triggering EMT and a more aggressive phenotype over time. The gained migration and invasion ability by long-exposed cells was associated with the increased expression level of key cellular and extracellular EMT-related factors. The PI3K/AKT pathway was a significant player in the acquired resistance of HCT-116 cells, possibly related to a *PI3KCA* mutation in this cell line. Our findings provide new insights into the phenotypic plasticity of CRC cells able, under treatment pressure, to acquire a stable TIS or to use an early senescence state to undergo EMT.

## 1. Introduction

Colorectal cancer (CRC) is one of the most frequent cancers and the third leading cause of cancer death worldwide [1]. Despite improvements over the last decade, patients with metastatic disease have a poor outcome [1,2]. Regorafenib (Stivarga^®^, Bayer AG, Leverkusen, Germany) was granted regulatory approval to treat patients with advanced, unresectable chemorefractory CRC (mCRC).

Regorafenib is a multikinase inhibitor (MKI) targeting tumour cells and their microenvironment by inhibition of several angiogenic RTKs (VEGFR-1, -2, -3, TIE-2), oncogenic RTKs (c-KIT, RET), stromal RTKs (PDGFR-B, FGFR1), intracellular signalling kinases (c-RAF/RAF-1, BRAF, BRAF^V600E^) and tumor immunity (CSF1R) [3,4]. Preclinical studies have demonstrated its anti-tumoural activity in several tumour models, including colorectal, gastrointestinal, and hepatocellular cancers [5]. Two phase III trials reported a significant, although limited survival advantage compared with a placebo [6,7]. More importantly, this treatment is associated with substantial toxicities, which limit its use in patients with poor general conditions and short life expectancy [4]. Hence, as the current conventional biomarkers and Response Evaluation Criteria in Solid Tumors (RECIST) are heavily limited by their lack of sensitivity [8,9,10,11], there is an urgent need for accurate biomarkers to detect non-responders to regorafenib therapy. However, given the complex interplay of the cellular pathways targeted by this drug, identifying related resistance mechanisms remains challenging.

Studies investigating mechanisms of therapy resistance have mainly focused on genetic drivers, such as gene alterations that prevent drug function or activate downstream effectors of alternative survival pathways [12,13]. Nevertheless, a literature review shows the relevance of non-mutational mechanisms as resistance determinants to targeted therapies. Indeed, surviving drug-tolerant tumour cells may acquire reversible or irreversible phenotypes in response to multiple extracellular signals. Tumour cell plasticity has been described as an evasion mechanism in numerous cancers [12,13].

In this context, therapy-induced senescence (TIS) has been characterised as a premature senescence mechanism induced by specific therapeutic agents. In addition to the loss of cell division ability, these cells are characterised by particular properties, such as alterations in cell morphology, metabolism, and their ‘omic’ landscape. They also exhibit profound changes in their secretome, named senescence-associated secretory profile (SASP), including a multitude of proteins (cleaved cell-surface molecules, growth factors, pro-inflammatory cytokines, and extracellular matrix-degrading enzymes) [14]. The subsequent molecular alterations allow cells to persist indefinitely with somewhat compromised viability, retaining the possibility of tumour regrowth in more favourable circumstances [15].

On the other hand, the initiation of epithelial-mesenchymal transition (EMT), due to exposure to anti-cancer therapeutic agents makes tumour cells more invasive and prone to metastasis [15,16]. These evolutionarily conserved processes play a role in tissue homeostasis and remodelling and have been investigated as cancer cell reprogramming models leading to drug-tolerant cell properties [12,13,15,17]. Cancer cell plasticity is characterised by cells able to interconvert several alternative states. SASP is a potential actor of cell plasticity as it modifies the tumour microenvironment to induce immune-mediated clearance of senescent cells or promote tumour progression [14]. In the present study, we explored the effects of short and long-term regorafenib exposure on representative human CRC cell lines to identify resistance mechanisms to this MKI.

## 2. Materials and Methods

### 2.1. Cell Lines and Cell Culture Conditions

Five human CRC cell lines, HCT-116 (ATCC^®^ CCL-247™), SW1116 (ATCC^®^ CCL-233™), LS-1034 (ATCC^®^ CRL-2158™), SW480 (ATCC^®^ CCL-228™) and Caco-2 (ATCC^®^ HTB-37™) were obtained from the American Type Culture Collection (ATCC, Manassas, VA, USA) and cultured according to the manufacturer’s recommendations. Cell line characteristics are described in Supplementary Table S1. HCT-116 cell line was grown in McCoy’s 5A Medium (ATCC^®^ 30-2007™), SW1116 in DMEM (Dulbecco/Vogt modified Eagle’s minimal essential medium), LS1034 in RPMI-1640 Medium (ATCC^®^ 30-2001), SW480 cells in Leibovitz’s L-15 Medium (ATCC^®^ 30-2008™) and Caco-2 in in Eagle’s Minimum Essential Medium (ATCC^®^ 30-2003) all supplemented with 10% fetal bovine serum (FBS) in addition to 100 U/mL of penicillin and 100 µg/mL of streptomycin at standard concentrations (all from Thermo Fischer Scientific Gibco and Invitrogen Waltham, MA, USA). HCT-116, SW1116, LS1034 and Caco-2 cell lines were cultured at 37 °C in a humidified 95% air with 5% CO_2_ atmosphere while the SW480 cell line was cultured at 37 °C without CO_2_.

### 2.2. Reagent

Regorafenib (BAY 73-4506) was purchased from Selleck Chemicals (Houston, TX, USA). Regorafenib powder was dissolved in DMSO according to the ‘manufacturer’s recommendation at 10^−2^ M, aliquoted and stored at −20 °C.

### 2.3. Cell Viability Assay

Crystal violet assay was used for cell proliferation viability assay. Cells were seeded in 96-well plates at cell-line-specific densities in a complete medium (Supplementary Table S1). The day after, the medium was replaced by fresh medium containing increasing regorafenib concentrations (2 × 10^−6^–2 × 10^−5^ M). After 72 h of treatment, the medium was removed. The cell layer was gently rinsed with phosphate-buffered saline (PBS) before fixation with 1% glutaraldehyde/PBS for 15 min and stained with 0.1% (*v*/*v* in water) crystal violet for 30 min. Finally, cells were washed with running tap water and afterward lysed with 0.2% (*v*/*v* in water) Triton X-100 for 90 min. The absorbance was measured at 570 nm using a Multiskan EX Microplate Photometer (Thermo Fischer Scientific, Waltham, MA, USA).

### 2.4. Development of Acquired Resistance to Regorafenib

To generate regorafenib-resistant cell lines, HCT-116 and SW480 parental cells were exposed to regorafenib at the starting concentration of 3 µM and 5.5 µM, respectively, and continuously treated for four weeks. Then, an increasing regorafenib concentration (by steps of 0.5 µM) up to 6 µM and 7 µM for HCT-116 and SW480 cell lines was applied for six months. Cells were kept under the last medium conditions for another six months (Rego-12m). Cell Line Authentication was performed using STR (Short Tandem Repeat) profiling with AmpFLSTR™ Identifiler™ PCR Amplification Kit (Thermo Fisher Scientific, Waltham, MA, USA). DNA isolation was conducted from a cell pellet of 10^6^ cells and 16 independent PCR-systems were investigated and analysed (Eurofins Genomics, Ebersberg, Germany) (Supplementary Table S2).

### 2.5. Detection and Measurement of β-Galactosidase Activity

The presence of Senescence-Associated (SA) β-galactosidase (SA-β-gal) was detected in situ using the β-Galactosidase Staining Kit (BioVision, Mountain View, CA, USA). Cells were plated in complete medium in 4-chamber slides (Thermo Fischer Scientific) at cell-line specific densities (Supplementary Table S1). The day after, the medium was replaced with a fresh one in the presence or absence of regorafenib according to the experimental conditions, and cells were cultured for 72 h. Then, cell monolayers were washed with PBS and fixed 15 min with the fixative solution at room temperature. After washing cells with PBS, the Staining Solution Mix containing 1 mg/mL X-gal (5-bromo-4-chloro-3-indolyl-beta-D-galactopyranoside) was applied to each chamber and cells were incubated for 24 h at 37 °C. Stained cells were visualised with the Vectra Polaris imaging system (Akoya Biosciences, Marlborough, MA, USA).

The β-gal activity was measured by flow cytometry as a substrate for beta-galactosidase the Fluorescein-di-beta-D-galactopyranoside (FDG) provided by the FluoReporter lacZ Flow Cytometry Kit F-1930 (Molecular Probe, Leiden, The Netherlands). Cells were plated in 6-well plates at cell-line specific densities (Supplementary Table S1) in a complete medium. The day after, the medium was replaced with a fresh one in the presence, or not, of regorafenib according to the experimental conditions. Cells were cultured for 72 h for both cell lines. After centrifugation, cell pellets were suspended in staining medium (PBS 4% (*v*/*v*) FBS and 10 mM HEPE at pH 7.2) containing FDG substrate and placed in 37 °C for 3 min. The enzymatic reaction stopped when 1.8 mL staining medium containing 1.5 μM propidium iodide (PI) were added. Cells were then examined in a flow cytometer (Beckman-Coulter Gallios 3L 10C, Beckman Coulter, Inc., Brea, CA, USA) and analysed with the Kaluza^®^ 1.3 Flow Analysis Software (Beckman Coulter, Inc.). Doublets were gated out and unstained control and control groups were used for Mean Fluorescence Intensity (MFI) evaluation of experimental conditions.

### 2.6. Cell Cycle Analysis

Cells were plated in 6-well plates at cell-line specific densities (Supplementary Table S1) in complete medium. The day after, the medium was replaced with a fresh one in the presence or absence of regorafenib according to the experimental conditions, and cells were cultured for 72 h. Cells were collected and fixed using 70% ethanol for at least 30 min on ice. After centrifugation, 400 µL of PI per 10^6^ cells were added and cells were incubated for 10 min at room temperature. Cell cycle analysis was performed by flow cytometry (Beckman-Coulter Gallios 3L 10C, Beckman Coulter, Inc.) and analysed with the Kaluza^®^ 1.3 Flow Analysis Software (Beckman Coulter, Inc.). The control group’s gating protocol was applied to each experimental condition.

### 2.7. RNA Isolation and Real-Time PCR

Total RNA was isolated from pellets of 10^7^ cells using the RNeasy Mini Kit (Qiagen, Valencia, CA, USA) and DNase I (Promega, Madison, WI, USA) according to the manufacturer’s instructions. Then, RNA concentration was assessed by the Qubit^®^ 2.0 fluorometer and the corresponding quantification kit (Qubit^®^ RNA BR Quantification Assay Kit (Thermo Fisher Scientific, Waltham, MA, USA). RNA (50 ng per sample) was reverse transcribed using a standard reverse transcription method (qScript cDNA SuperMix, Quanta Biosciences, Gaithersburg, MD, USA). cDNA was amplified by RT-PCR using Power SYBR Green PCR Master Mix (Applied Biosystems, Carlsbad, CA, USA) and specific primers (Supplementary Table S3) for the following genes: *p16*, *p19*, *p27*, *TGF-β*, *ZEB1*, *MMP9*, *N-Cad*, and *18S*. The relative quantification of mRNA was determined by normalizing the crossing threshold (CT) of analysed genes with the CT of 18S (housekeeping gene) and using the 2^−ΔΔCT^ method. Triplicates were used for each sample, and each group was analysed three times.

### 2.8. Western Blot Analysis

Total proteins were extracted from cells treated or not with regorafenib using the M-PER™ Mammalian Protein Extraction Reagent kit in association with Halt™ Protease and Phosphatase Inhibitor Cocktail, EDTA-free (1/100), all from Thermo Fischer Scientific, Waltham, MA, USA. Using bovine serum albumin as the standard, protein quantification was determined with the Pierce™ BCA Protein Assay (Thermo Fischer Scientific, Waltham, MA, USA). Equal amounts of extracted proteins (35 µg) were loaded in 4–20% Mini-PROTEAN^®^ TGX™ Precast Gels (Bio-Rad, Hercules, CA, USA) and electrotransferred to nitrocellulose membranes using an iBlot^®^ Dry Blotting System (Invitrogen, Life Technologies, Gent, Belgium). Immunodetections were performed using primary antibodies raised against the following proteins: AKT (pan) (1/1000, cat. No. 2920), phospho-AKT(Ser473) (1/2000, cat. No. 4060), ZEB1 (D80D3) (1/000, cat. No. 3396), E-Cadherin (24E10) (1/1000, cat. No. 3195), β-Actin (D6A8) (1/1000, cat. No. 8457) all from Cell Signaling Technology, Danvers, MA, USA; ERK2 (1/2000, cat. No. sc154), phosphor-ERK (Tyr 204) (1/1000, cat. No. sc7383) from Santa Cruz Biotechnology (Santa Cruz, Dallas, TX, USA) and Cyclin A2 antibody [E23.1] (1/200, cat. No. ab38) from Abcam, Cambridge, UK. The antibodies were incubated with the membranes at room temperature for 1 h and then at 4 °C overnight. Peroxidase-labelled anti-rabbit (cat. No. NA931) or anti-mouse (cat. No. NA931) IgG antibodies (1/5000) (Amersham Pharmacia Biotech, Roosendaal, The Netherlands) were incubated with the membranes at 4 °C for 1 h to detect corresponding primary antibodies. Then, SuperSignal^®^ West Pico Chemiluminescent Substrate (Thermo Fischer Scientific, Waltham, MA, USA) was used to reveal peroxidase activity following manufacturer’s instruction. Immunostaining signals were visualised with a PC-driven LAS-3000 charge-coupled device (CCD) camera (Fujifilm, Tokyo, Japan), via an image acquisition software (Image Reader, Raytest^®^, Straubenhardt, Germany). The intensities of detected signal bands were quantified using the software ImageJ.

### 2.9. Enzyme Linked Immunosorbent Assay (ELISA)

The Quantikine^®^ Human Immunoassays (all from R&D Systems, Inc., Minnesota, CO, USA) were used to quantify soluble AXL (cat. No. DAXL00), ICAM1 (cat. No. DCD540), IL6Rα (cat. No. DR600) and IL8 (cat. No. D8000C) in cell culture supernatants from treated or not cell lines, according to manufacturer’s instructions. The optical density per well was measured at 450 nm and 570 nm to correct optical imperfections using a Multiskan EX Microplate Photometer (Thermo Scientific, Courtaboeuf Cedex, France).

### 2.10. Cell Migration Assay

Cell migration assay (HCT-116; 3000 cells/insert, SW480; 10,000 cell/insert) was performed using Transwell^®^ polycarbonate membrane inserts with 8.0 µm pores (Corning, Incorporated, NY, USA). Serum-free medium-containing cells were seeded in cell culture inserts. Then, inserts were put in a 24 well-plate containing complete medium. After 24 h, insert media were replaced with new serum-free media containing or not regorafenib according to the experimental conditions and further incubated for 72 h. After removal from the inner part, cells were stained with a 0.1% crystal violet solution. Five random fields were counted for each group (magnification ×10) using Zeiss Axio scope A1 microscope (Carl Zeiss, Oberkochen, Germany). Cell counts were determined with ImageJ software.

### 2.11. Cell Invasion Assay

For the cell invasion assay (HCT-116; 6000 cells/insert), Corning^®^ BioCoat™ Matrigel^®^ Invasion Chambers with 8.0 µm PET Membrane were used (Corning, Incorporated, NY, USA). Serum-free medium-containing cells were seeded in cell culture inserts. Then, inserts were put in a 24 well plate containing complete medium. After 24 h, insert media were replaced with new serum-free media containing or not regorafenib according to the experimental conditions and further incubated for 72 h. After removal from the inner part, cells were stained with a 0.1% crystal violet solution. Five random fields were counted for each group (magnification ×10) using Zeiss Axio scope A1 microscope (Carl Zeiss, Oberkochen, Germany). Cell counts were determined with ImageJ software.

### 2.12. Immunofluorescence Microscopy

Cells were plated in complete medium in 4-chamber slides (Thermo Fischer Scientific) (6 × 10^3^ and 20 × 10^3^ cells/chamber for HCT-116 and SW480 cell lines, respectively). After 24 h, the medium was replaced with a fresh one in the presence or absence of regorafenib according to the experimental conditions, and cells were cultured for 72 h. Then, cell monolayers were fixed with the Image-iT™ Fixative Solution (4% formaldehyde, methanol-free) (ThermoFischer Scientific, Waltham, MA, USA). Before antibody exposure, cells were washed several times with DPBS containing 0.1% Triton X-100 and blocked for 30 min with DBPS containing 1% BSA. Primary antibodies were used against Phospho-Histone H3 (Ser10) (1/500, cat. No. PA5-17869, ThermoFischer Scientific, Waltham, MA, USA), E-Cadherin (24E10) (1/200, cat. No. #3195, Cell Signaling Technology, Danvers, MA, USA) and were exposed 2 h at room temperature. Cells were rinsed and incubated for 1 h with the secondary antibody Goat anti-Rabbit IgG (DyLight 488, 2 µg/mL, cat. No. 35552, ThermoFischer Scientific, Waltham, MA, USA). Samples were mounted with Vectashield Antifade Mounting Medium with DAPI to stain the nucleus (Cat. No. H-1200-10, Vector Laboratories, Newark, CA, USA). Stained and unstained cells (used as a control for background noise) were visualised with the Vectra Polaris imaging system (Akoya Biosciences, Marlborough, MA, USA). Five random fields were counted for each sample, and images were analysed with inForm Tissue Finder software (Akoya Biosciences).

### 2.13. Statistical Analysis

Regorafenib concentration inhibiting 50% of cell growth (IC_50_) was evaluated using dose-response curves from the GraphPad Prism software version 7 (LA Jolla, CA, USA). Gene expression data were expressed as means ± SEM of at least three independent experiments. The statistical significance was assessed by Student’s *t*-test (GraphPad software, LA Jolla, CA, USA), and multiple comparisons were corrected with the Holm-Sidak method. *p*-value < 0.05 was considered as statistically significant and classified as following: * *p* < 0.05, ** *p* < 0.01, *** *p* < 0.001.

## 3. Results

### 3.1. Short-Term Treatment with Regorafenib Affects CRC Cell Survival and Signalling Pathways

**Figure 1:**
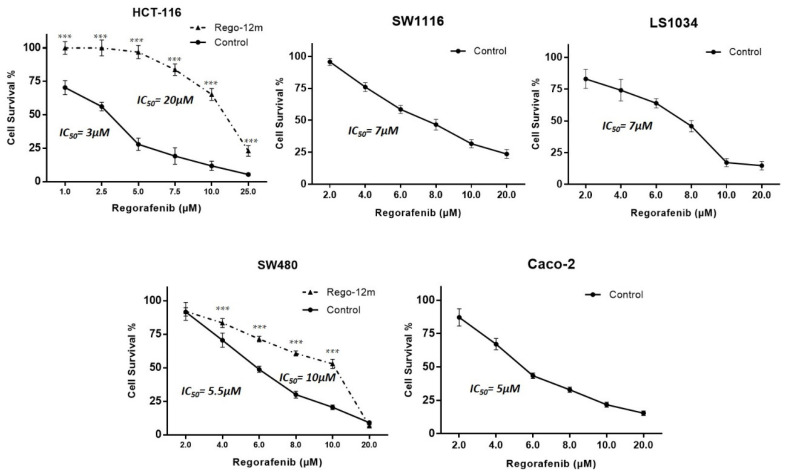
Regorafenib effect on survival of HCT-116, SW1116, LS1034, SW480 and Caco-2 cell lines. Proportion of surviving cells under regorafenib assessed by crystal violet. Data are expressed as means ± SEM (n ≥ 3) compared to control group with *** *p* < 0.001 values from Student’s *t*-test.

The proportion of surviving HCT-116, SW1116, LS-1034, SW480, and Caco-2 parental (control) cells was evaluated under different drug concentrations to determine half-maximal inhibitory concentrations (IC_50_). IC_50_ values were assessed after three days of treatment with 3 µM, 7 µM, 7 µM, 5.5 µM and 5 µM for HCT-116, SW1116, LS-1034, SW480 and Caco-2 cells, respectively (Figure 1).

**Figure 2:**
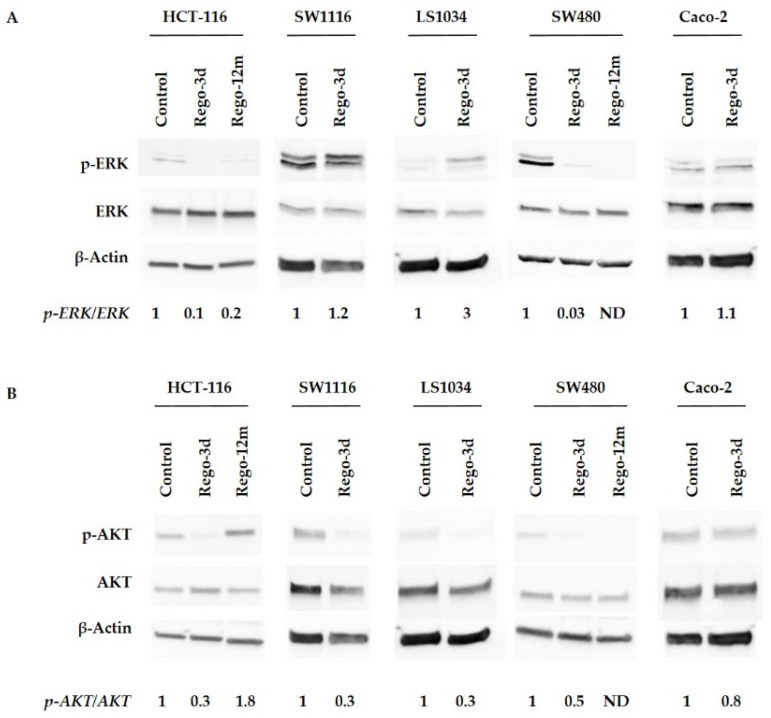
Regorafenib effect on ERK (**A**), AKT (**B**) signaling pathways of HCT-116, SW1116, LS1034, SW480 and Caco-2 cell lines. ERK and AKT phosphorylation levels assessed by Western blotting and related fold changes. β-Actin is used as a loading control. Control cells; Rego-3d (short regorafenib exposure (3 days)); Rego-12m (long regorafenib exposure (12 months)); p-ERK (phosphorylated ERK); p-AKT (phosphorylated-AKT); ND (not detectable).

Then, we investigated the effects of regorafenib on the MAPK and PI3K/AKT cell signalling pathways, the two main mechanisms controlling cell proliferation and survival, by assessing the phosphorylation of ERK (p-ERK) and AKT (p-AKT). An apparent decrease in p-ERK levels was observed after short exposure (Rego-3d) in HCT-116 and SW480 cell lines, while no difference was observed for SW1116 and Caco-2 cells. Phosphorylation levels of ERK in LS1034 cell lines were increased after short exposure to regorafenib (Figure 2A). Regarding p-AKT, protein levels decreased in all cell lines after three days of exposure except in Caco-2 cell line where levels remained similar (Figure 2B).

### 3.2. Short-Term Regorafenib Exposure Induces Early Senescence-Like Properties

**Figure 3:**
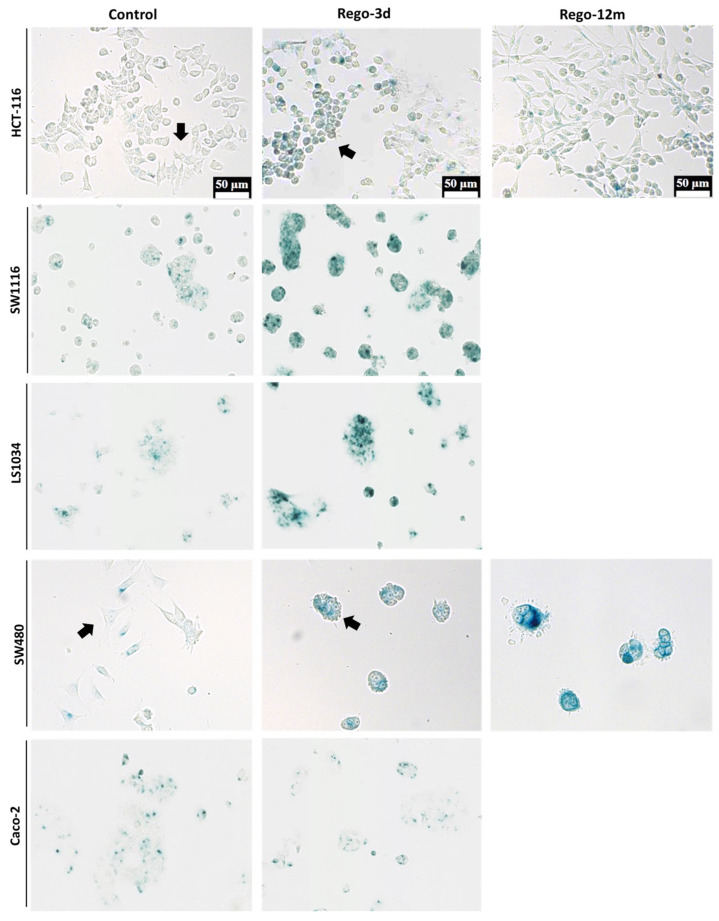
Detection of Senescence-Associated β-galactosidase (SA-β-Gal) in HCT-116, SW1116, LS1034, SW480 and Caco-2 cell lines after Regorafenib exposure. Representative sections of SA-β-Gal activity by in situ staining (in blue). Representative cells with different shape (Control vs Rego-3d group) are marked with arrows. Control; Rego-3d (short regorafenib exposure (3 days)); Rego-12m (long regorafenib exposure (12 months)).

An irregular cell morphology (higher cell volume, different cell shape), more pronounced in SW480 and HCT-116 cell lines (Figure 3), was noticed while observing day-by-day CRC cell lines under regorafenib. This characteristic prompted us to investigate senescence markers to determine a potential phenotype switch following regorafenib exposure.

**Figure 4:**
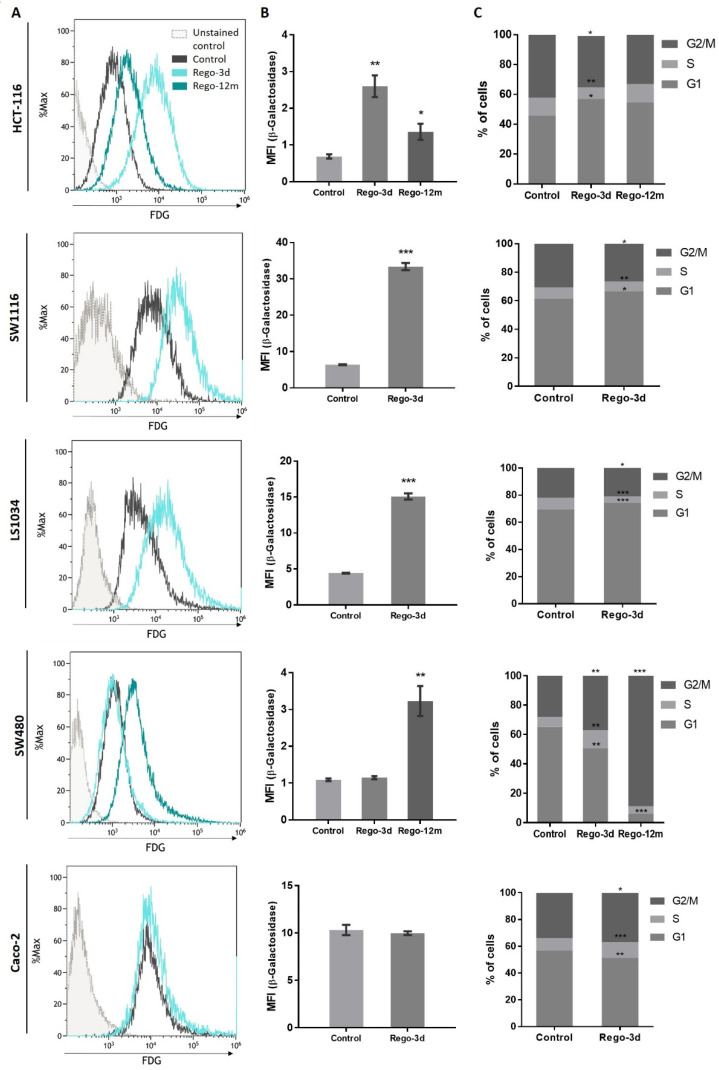
Regorafenib effect on Senescence-Associated β-galactosidase (SA-β-Gal) activity and cell cycle in in HCT-116, SW1116, LS1034, SW480 and Caco-2 cell lines after Regorafenib exposure. (**A**,**B**) SA-β-Gal activity assessment by detecting the SA-β-Gal substrate (fluorescein-di-beta-D-galactopyranoside (FDG)) by flow cytometry. (**C**) Proportion of cells detected in G0/G1, S and G2/M phase by flow cytometry through DNA staining (propidium iodide). Control; Rego-3d (short regorafenib exposure (3 days)); Rego-12m (long regorafenib exposure (12 months)). Data are expressed as means ± SEM (n ≥ 3) compared to control group with * *p* < 0.05, ** *p* < 0.01, *** *p* < 0.001 values from Student’s *t*-test.

The cytochemical detection of the Senescence-Associated β-galactosidase (SA-β-Gal), a marker of cellular senescence, showed that all control cells presented low SA-β-Gal staining. After a short exposure to regorafenib (Rego-3d), levels of SA-β-Gal increased in HCT116, SW1116, and LS1034 cell lines, while a slight increase was observed in SW480 cells, and similar levels were obtained for Caco-2 treated cells (Figure 3). In line with these results, when compared to control cells, short-term treated cells showed a statistically higher SA-β-Gal activity measured by flow cytometry in HCT-116, SW1116 and LS1034 cell lines (3 to 4.5-fold increase, *p* < 0.01) (Figure 4A,B).

Following these indications, we aimed to identify changes in the proportion of cells in the cell cycle phases through DNA staining (propidium iodide) using flow cytometry. After a short exposure to regorafenib (Rego-3d), we observed a statistically significant increase of cell proportion in the G1 phase and a decrease in the G2 phase for HCT-116, SW1116 and LS1034 cell lines. However, the opposite was observed in SW480 and Caco-2 treated cells (cell proportion decreased in G1 and increased in G2) when compared to the control group (Figure 4C).

### 3.3. Induction of Long-Term Acquired Resistance to Regorafenib

These preliminary findings led to the evaluation of CRC phenotype switching as a resistance mechanism to regorafenib. To better understand its long-term implementation and the potential link with acquired resistance to treatment, HCT-116, and SW480 cell lines were selected and used as models. In fact, when compared to the other studied cell lines, both of them demonstrated morphologic changes, an increase in SA-β-Gal staining or activity (Figure 3 and Figure 4A,B), and presented a higher difference in cell proportion arrested in G1 or G2 phase, respectively (Figure 4C), after short exposure to regorafenib. Taken together, these preliminary results led us to investigate further a drug-initiated senescence-like phenotype as a cell death escape mechanism in these two cell lines showing the more obvious changes under regorafenib.

To develop a potential acquired resistance, cells were treated at a starting concentration of IC_50_ values of control cells up to 6 µM and 7 µM for HCT-116 and SW480 cell lines, respectively, during 12 months (Rego-12m group). By comparing surviving curves of control cells and long-term treated cells (Rego-12m group), we observed a higher degree of resistance to regorafenib as IC_50_ value rose to 20 µM in HCT116 Rego-12m group (6,6-fold increase) and 10 µM (1.8-fold increase) in SW480 Rego-12m group (Figure 1). Of note, the STR profiling did not reveal any cross-contamination between cell lines and confirmed the identical genetic identity between the control and long-term treated group for both cell lines (Supplementary Table S2).

### 3.4. Long Exposure to Regorafenib Induces a Stable TIS Phenotype in SW480 Cells

Compared to control cells, a 3-fold decrease (*p* = 0.02) in cell growth was observed in SW480 long-term treated cells (Figure S1). In line with this data, the phosphorylation levels of ERK and AKT dropped after prolonged exposure to regorafenib compared to control or Rego-3d cells (Figure 2). Moreover, an important increase of SA-β-Gal staining (Figure 3) and activity (3-fold increase, *p* < 0.01) (Figure 4) was observed after 12 months of exposure.

**Figure 5:**
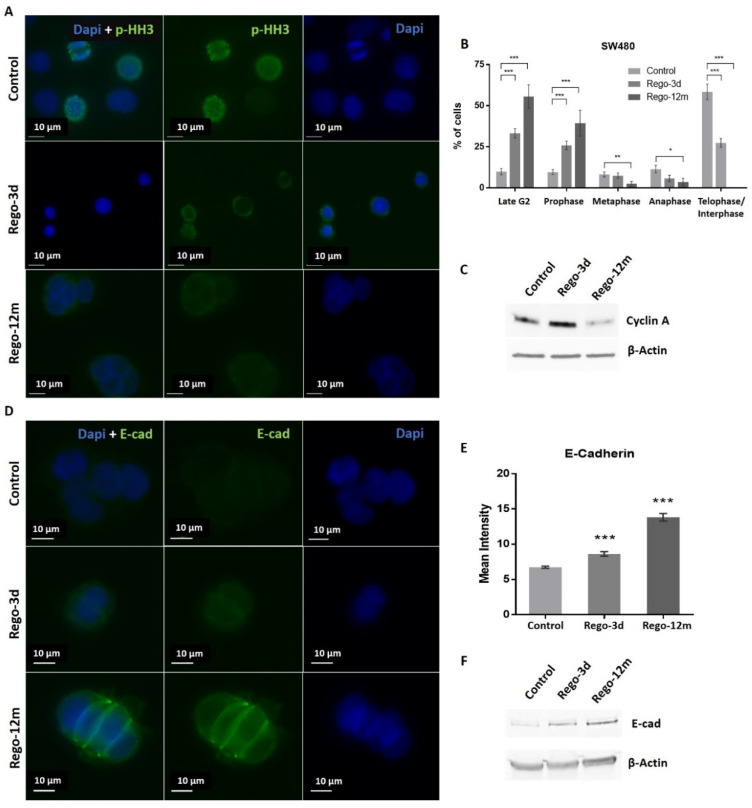
Regorafenib effect on cell division stages and adhesion in SW480 cell line. (**A**) Representative sections of labelled nuclei using p-HH3 (in green) and Dapi staining (in blue). (**B**) Column diagram indicating proportion of cells per cell division phase according to phospho-histone H3 (p-HH3) nuclear staining detected by immunofluorescence. Cells without p-HH3 staining are gathered in the telophase-interphase group. (**C**) Cyclin A levels assessed by Western blotting. β-Actin is used as loading control. (**D**) Representative sections of labelled cell surface using E-cad (in green) and cell nuclei using Dapi staining (in blue). (**E**) Quantification of E-Cadherin (E-cad) expression based on fluorescence density quantified with ImageJ software and normalised to cell number. (**F**) E-cad protein levels assessed by Western blotting. β-Actin is used as loading control. Control; Rego-3d (short regorafenib exposure (3 days)); Rego-12m (long regorafenib exposure (12 months)). Data are expressed as means ± SEM (n ≥ 3) compared to control group with * *p* < 0.05, ** *p* < 0.01, *** *p* < 0.001 values from Student’s *t*-test.

Regarding cell cycle analysis, results have shown that the majority of long-term treated cells were arrested in the G2/M phase (Rego-12m, 80.4%) as compared to the control group (G2/M, 18.6%; *p* < 0.001) (Figure 4C). We then performed a deeper cell cycle distribution analysis using histone H3 phosphorylation (p-HH3) as a mitosis marker also observable at the late G2 phase. While P-HH3 localization in the nucleus is an indicator of mitosis, its expression is not noticeable at the end of mitosis (telophase) and during the interphase [18,19]. Our data show a significantly decreased percentage of mitotic cells in metaphase/anaphase (*p* < 0.05) as well as a significantly increased proportion of treated cells in late G2 and prophase over time (*p* < 0.001), suggesting a late G2 phase or prophase arrest rather than late mitotic phase arrest (Figure 5A,B). This result is also supported by the remarkably lower proportion of long-term treated cells in telophase/interphase (*p* < 0.001) and the decreasing protein expression level of Cyclin-A (highly expressed in early G2 phase) in the Rego-12m group as compared to control cells (Figure 5B,C).

In addition, we noticed a change in cell behaviour with the formation of cell clusters. Accordingly, we observed a significant increase in the E-cadherin (E-cad) protein expression level in cells exposed to regorafenib (2-fold increase in Rego-12m group, *p* < 0.001) (Figure 5D–F). These data indicate a higher cell adhesion ability under treatment, which may also characterize a senescence-like phenotype [20].

**Figure 6:**
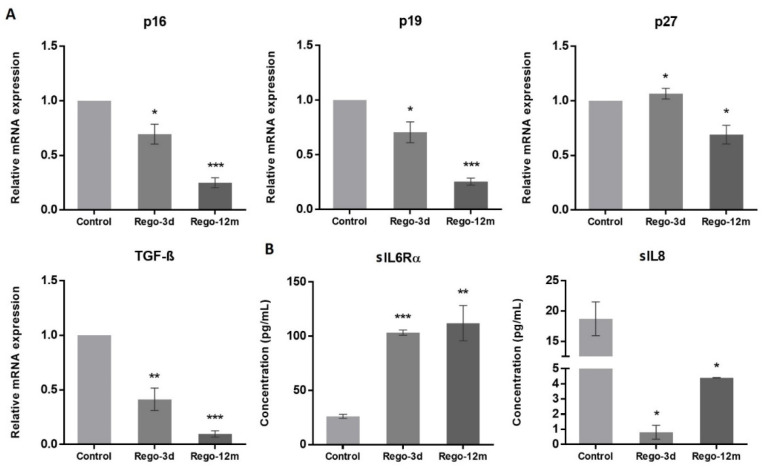
The effect of regorafenib on cell cycle regulators and senescence-associated secretory phenotype in SW480 cell line. (**A**) Relative gene expression involved in cell cycle regulation of SW480 cells determined by real-time PCR and presented as 2^−ΔΔCT^ values and (**B**) Soluble (s) protein quantification into respective cell supernatants using ELISA. Control, Parental cells; Rego-3d, short regorafenib exposure (3 days), Rego-12m, long regorafenib exposure (12 months). Data are expressed as means ± SEM (n ≥ 3) compared to control group with * *p* < 0.05, ** *p* < 0.01, *** *p* < 0.001 values from Student’s *t*-test.

At a molecular level, we observed a statistically significant decrease in the relative expression levels of G1 Cyclin-Dependent Kinase (CDK) inhibitors and key cell cycle regulators in treated cells and more particularly in the Rego-12m group (p16, p19, TGF-β; *p* < 0.001 and p27; *p* < 0.05) (Figure 6A). Altogether, these results suggest a preferential cell cycle arrest in the G2 late phase and mitosis prophase stage after prolonged regorafenib exposure.

Notably, a lower proportion of migratory cells was observed under regorafenib exposure compared to control cells. More precisely, no cell migrated in the Rego-12m group, possibly due to a critical decrease of ZEB1 gene and protein expression levels in this condition as compared to the control group (Figure S1B–E).

As senescent cells exhibit a specific secretory phenotype called Senescence-Associated Secretome (SAS), we assessed some reported factors in the culture media of control and treated cells [21]. We found a significantly higher concentration of soluble (s) interleukin 6 receptor α (sIL6Rα) in supernatants of treated cells (Rego-3d, *p* < 0.001; Rego-12m, *p* < 0.01), while soluble interleukin 8 (sIL8) concentration was significantly lower as compared to control cells (Rego-3d and Rego-12m; *p* < 0.05) (Figure 6B). These data suggest a cell secretome dysregulation under regorafenib exposure in a time-dependent manner.

### 3.5. Long-Term Regorafenib Treatment Induces an Early Senescence-Like State to Undergo EMT in HCT-116 Cells

#### Cell Senescence-Like Features induced by Regorafenib

In HCT-116 cell line, an apparent decrease in p-ERK levels was observed after prolonged exposure, while p-AKT increased considerably compared to the control group suggesting the potential involvement of AKT activation in the related acquired resistance (Figure 2).

The investigation of senescence markers in HCT-116 cells revealed the presence of some senescence properties after regorafenib long exposure, such as the significant increase in SA-β-Gal staining and activity in Rego-12m cells (2-fold increase, *p* < 0.05) as compared to control cells (Figure 3 and Figure 4A,B).

**Figure 7:**
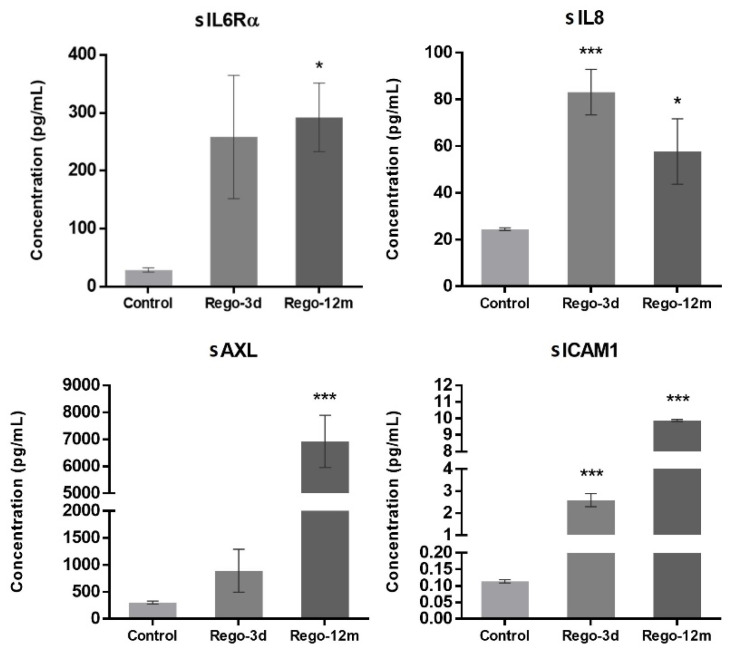
Regorafenib effect on cell secretome of the HCT-116 cell line. Soluble (s) protein quantification into respective cell supernatants using ELISA. Control; Rego-3d (short regorafenib exposure (3 days)); Rego-12m (long regorafenib exposure (12 months)). Data are expressed as means ± SEM (n ≥ 3) compared to control group with * *p* < 0.05, *** *p* < 0.001 values from Student’s *t*-test.3.5.2. Regorafenib Effect on EMT, Cell Migration, and Invasion.

In contrast to short-term treated cells, the cell cycle analysis of HCT-116 long-term treated cells did not reveal any statistical difference among the proportion of cells in a specific stage compared to the control group (Figure 4C). However, we observed higher levels of sIL6Rα and sIL8 levels after both short- (sIL8, *p* < 0.001) and long-term (sIL6Rα and sIL8, *p* < 0.05) exposure to regorafenib (Figure 7), suggesting a secretome dysregulation of treated cells in favor of a senescence-like phenotype. Of note, while sIL6Rα was upregulated in both SW480 and HCT-116 lines, sIL8 was downregulated in the former line and upregulated in the latter.

**Figure 8:**
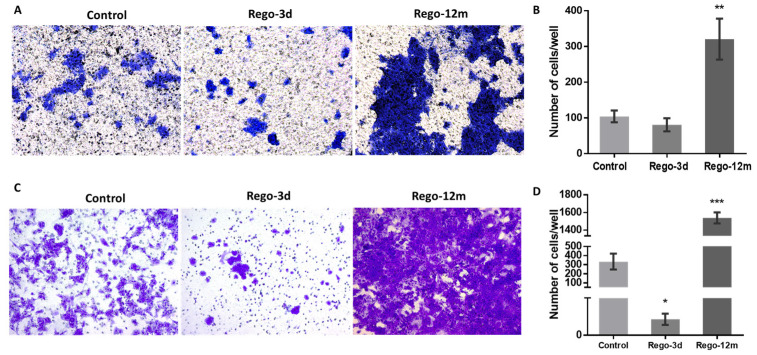
Regorafenib effect on migration and invasion ability in HCT-116 cell line. Representative pictures showing migration (**A**) and invasion (**C**) ability of cells in Control; Rego-3d (short regorafenib exposure (3 days)) and Rego-12m groups (long regorafenib exposure (12 months)) (crystal violet staining, 10× magnification). Column diagram indicating the amounts of migrating (**B**) and invading (**D**) cells per field. Data are expressed as means ± SEM (n ≥ 3) compared to control group with * *p* < 0.05, ** *p* < 0.01, *** *p* < 0.001 values from Student’s *t*-test.

Bearing in mind that some factors secreted by senescent cells, and more precisely the IL8 chemokine, may induce EMT and invasiveness [15,21], we explored whether regorafenib treatment could affect the EMT of HCT-116 cells. Firstly, treated cell’ migration and invasion ability were investigated and compared to controls. Higher proportions of migratory (Figure 8A,B, *p* < 0.01) and invasive (Figure 8C,D, *p* < 0.001) cells were significantly observed in the long-term treated group. Moreover, short spindle-shaped cells were also observed in the Rego-12m group compared to the control cells suggesting an acquired mesenchymal phenotype (Figure 3).

**Figure 9:**
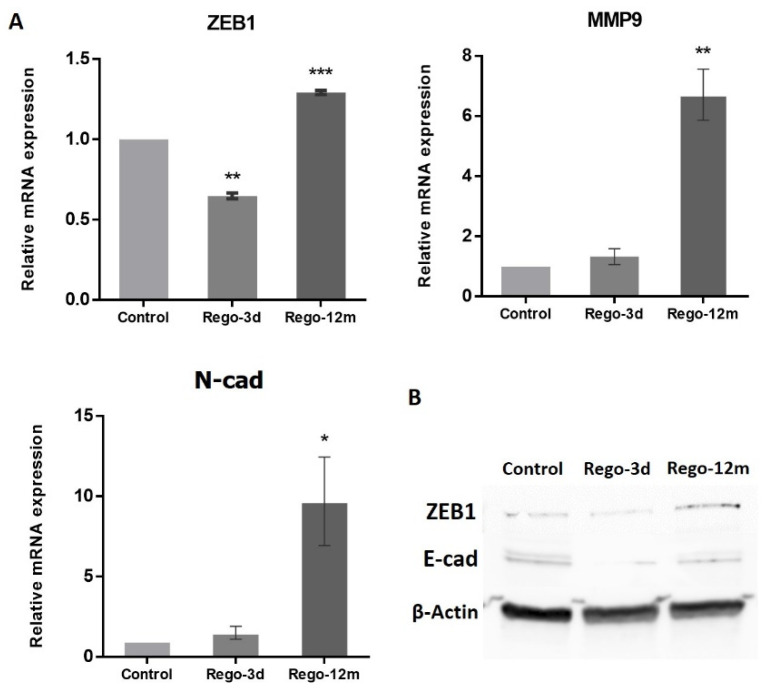
Regorafenib effect on Epithelial-Mesenchymal Transition (EMT) in the HCT-116 cell line. (**A**) Relative gene expression involved in EMT phenotype of HCT-116 cells determined by real-time PCR and presented as 2^−ΔΔCT^ values. (**B**) ZEB1 and E-cad protein levels assessed by Western blotting. β-Actin is used as loading control. Control; Rego-3d (short regorafenib exposure (3 days)); Rego-12m (long regorafenib exposure (12 months)). Data are expressed as means ± SEM (n ≥ 3) compared to control group with * *p* < 0.05, ** *p* < 0.01, *** *p* < 0.001 values from Student’s *t*-test.

At a molecular level, the migration ability gained by the Rego-12m HCT-116 cells was associated with the increased expression level of key EMT factors. Genes such as *ZEB1* (*p* < 0.001), *MMP9* (*p* < 0.01), and *N-cad* (*p* < 0.05) were upregulated in Rego-12m cells as compared to control cells (Figure 9). Interestingly, the gene and protein expression level of ZEB1 was significantly downregulated after short exposure as compared to the control group (Figure 9A,B, *p* < 0.01), which could explain the less invasive profile of cells treated for three days (Figure 8C,D). Likewise, the protein expression level of E-Cad (epithelial cell marker) was decreased in treated cells (Figure 9B).

Furthermore, the concentration of two secreted proteins involved in the EMT process, sAXL and sICAM1, were significantly increased in cell supernatants of treated cells (Rego-12m: *p* < 0.001) in comparison with control cells (Figure 7), suggesting the acquisition of a mesenchymal phenotype.

## 4. Discussion

The intrinsic and acquired resistance mechanisms to regorafenib were investigated after short- and long-term exposure on five (HCT-116, SW1116, LS-1034, SW480, Caco-2) and two (HCT-116, SW480) CRC cell lines, respectively. This study revealed that regorafenib treatment induces a phenotype switch in some CRC cell lines with senescence-like properties, potentially leading to different acquired resistance mechanisms.

The anti-tumoral activity of regorafenib is based on combined antiangiogenic and anti-stromal effects [5]. We show a substantial inhibition of p-ERK after short exposure, mainly in HCT-116 and SW480 cell lines in line with the existing literature supporting an upstream inhibition of VEGFRs, BRAF, KIT, and TIE-2 kinases [5,22,23,24,25]. Moreover, the PI3K/AKT pathway was similarly affected after short-term exposure in all cell lines, with a significant decrease in p-AKT expression levels. However, while in SW480 long-term treated cells, p-AKT level remained much lower than in control cells, the opposite was found for HCT-116 treated cells suggesting an acquired resistance mechanism exploiting the PI3K/AKT pathway. Of note, the *PI3KCA* gene mutation in this cell line could explain the persistence of AKT phosphorylation after long treatment exposure. The impact of regorafenib on ERK and AKT phosphorylation levels has already been investigated after short-term exposure (24 h) in CRC lines, and reported data globally show a decrease compared to non-treated cells [5,23,24,25,26,27]. In contrast, data on “regorafenib-resistant” generated cells are more difficult to interpret given the heterogeneity between studies regarding exposure time and drug concentration that could impact the establishment of a potential resistance mechanism through the activation of the PI3K/AKT pathway [24,26,28].

The observation of morphological changes, a higher SA-β-Gal staining/activity and different cell proportions in the cell cycle phases after short exposure in most cells led us to investigate a drug-initiated senescence-like phenotype as a cell death escape mechanism widely associated with resistance to treatment. To explore this phenotype switch’s presence following long-term regorafenib treatment, HCT-116, and SW480 cell lines were used as models, and additional markers were evaluated. These two cell lines were selected for long-term investigation as they presented more obvious changes in their phenotype after short exposure to regorafenib. Exposing cells to low cytotoxic concentrations of regorafenib for an extended period (12 months) generated a higher degree of acquired resistance in HCT-116 cell line than in the SW480 cell line. At the same time, the former was more sensitive in the control group.

The first resistance mechanism that we encountered in our experiments is a stable therapy-induced senescence-like (TIS) phenotype in the SW480 CRC cell line, continuing throughout long-term exposure. Several studies have demonstrated TIS as a resistance mechanism, including CRC in vitro and in vivo models under chemotherapeutic agents [16,29,30]. These studies highlighted cells displaying a specific phenotype with senescence-like cell features after exposure to radiation and/or chemotherapeutic drugs [16,30,31].

Under regorafenib, SW480 cells trigger a senescent-like phenotype characterised by a shallow growth rate, altered morphology, and a gradually increasing level and intensity of SA-β-Gal activity over time. A different cell behaviour was also observed with an increased E-cad expression, supported by the downregulation of *ZEB1* and *TGF-β* genes, leading to a noticeable formation of cell colonies [20,32,33]. A preferential cell growth arrest in the late G2 phase, led by CDK inhibitors’ downregulation of the G1 phase (*p16* and *p19*) and the downregulation of *p27* and *TGF-β* (which regulates as well p15 and p21), supports the TIS phenotype [34,35]. The expression levels of P-HH3 and Cyclin A, markers of mitosis and S/early G2 phase, respectively, also suggested a higher proportion of arrested long-term treated cells in late G2 and early mitotic phase (prophase). The almost complete cell cycle arrest induced by regorafenib after long exposure is also sustained by the dramatic reduction of p-AKT in these cells. In keeping with our findings in the SW480 “*p53* inactive” cell line, it has been reported that p53 knockout can induce senescence rather than suppress the TIS response induced by chemotherapeutic agents in cancer cells [30], suggesting that the stable senescence that we observed in this cell line could be linked to the *p53* mutation. A cancer cell phenotypic change to a stable “dormant” state under treatment may potentially have meaningful consequences in clinical practice, as TIS provides these cells with a death-escaping mechanism potentially persisting throughout several therapy cycles [36]. However, under appropriate conditions, such as decreased drug-concentration exposure or acquired resistance mechanisms, these “dormant” cells may “wake up” and contribute to disease recurrence [36,37,38].

A second acquired resistance mechanism has been identified in HCT-116 cell line through cell plasticity involving two phenotypes. On the one hand, our data revealed induction of senescence features under regorafenib at SA-β-gal level, and activity was increased in treated cells (mainly after short-term exposure), however, without necessarily exhibiting an evident growth arrest contrary to SW480 treated cells. Indeed, compared to control cells, the long-exposed condition did not present any statistical difference in cell cycle analysis in contrast with short-term exposure, where a lower proportion of HCT-116 cells in the S and G2 cell-cycle phases was observed. In the literature, a decreased proportion of cancer cells in the S phase under anti-cancer therapy has already been reported [30]. Additionally, Li-Ching and colleagues found a lower Cyclin D1 protein level in HCT-116 cells after 24 h of regorafenib exposure, suggesting a preferential arrest in the early interphase stage [39]. Together, these results indicate that the HCT-116 cell line has cellular plasticity allowing the acquisition of senescence-like features shortly after regorafenib exposure.

The senescence process is characterised by the secretion of several proteins, known as senescence-associated secretome (SAS), that include cytokines, chemokines, and proteases having substantial effects on stromal cells through autocrine and paracrine mechanisms [21]. IL6 plays a central role in the senescence/inflammatory response by binding to its membrane receptor (mIL-6R) [21,40]. In intestinal cells, the activation of intracellular pathways such as STAT3 does not depend only on the expression of membrane IL6R but also relies on IL6 trans-signalling through the soluble form of IL6R (sIL6R) [40,41]. In this context, we have demonstrated that in both cell lines, after short and long regorafenib exposure, cells secreted sIL6Rα to high levels, further supporting the contention that this pathway is essential to initiate and maintain senescence-associated properties [42]. Overall, these data reinforce our findings of a TIS phenotype of SW480 treated cells. In addition, regarding the HCT-116 cell line, Niu and colleagues have recently reported that the SAS-related p16/IL6 axis, in parallel with an AKT phosphorylation activation, contributes to the formation of acquired resistance in hepatocellular carcinoma cells receiving long-term exposure to sorafenib, another MKI [43]. Likewise, the IL8-IL8R axis has been shown to play a role in the senescence process and impact tumour growth, metastasis, and angiogenesis in CRC [21,41]. In supernatants of HCT-116 cells exposed to regorafenib, high amounts of sIL8 were observed. However, SW480 cells treated in the same conditions presented an essential drop in sIL8, suggesting that, in this case, the IL8-IL8R axis may not be necessary to initiate or preserve the stable TIS phenotype. Our findings support that tumour cellular senescence response after regorafenib exposure is associated with mediators known to play a role in senescence-like phenotype switching [44].

Considering that cellular senescence can trigger tumourigenesis and create a microenvironment for metastasis by enhancing SAS, especially IL8 cytokine, we investigated the migratory and invasive capacity of HCT-116 treated cells [17,45,46]. We observed that, compared to control cells, long treatment exposure primarily increased (3-fold increase) the migratory ability of cells and (10-fold increase) their invasion capacity. These data align with the increasing amounts of sIL8, sICAM1, and sAXL in supernatants of long-term exposed cells, as these factors have been associated with an EMT state, a mesenchymal phenotype, an advanced CRC stage, or short patient survival [41,47,48]. Moreover, the high levels of p-AKT and the upregulation of *ZEB1*, *MMP9*, *N-cad* gene expression, and the downregulation of E-cad protein expression after long-term regorafenib exposure support our finding of a more important migratory and invasive ability. These results highlight the respective role of these EMT-associated factors in tumour progression. At the same time, the downregulation of *ZEB1* is likely related to the described senescent properties of cells after short drug exposure [15,49]. Likewise, Tomida and colleagues have shown that 90 days of regorafenib exposure dramatically increase migration and invasion activities via resistance to the hypoxia-induced apoptosis mechanism in HCT-116 cell line [50]. They suggested that the loss of VEGF/VEGFR signalling in tumour cells contributes to the acquired resistance of VEGFR-targeted therapies via activation of c-Met, STAT3, and PI3K/AKT signalling pathways [50].

Our findings on HCT-116 cell line suggest that regorafenib long exposure promotes the co-existence of senescent and EMT phenotypes. Indeed, the presence of a SAS, including inflammatory compounds, may promote tumour growth and angiogenesis, leading to a simultaneous appearance of cells presenting EMT properties [15,51,52,53].

Altogether, our observations support the widely accepted view that MKI-induced resistance phenotype may be initiated and maintained in a subpopulation of cells with senescent-like properties [36,42,45,54]. Despite the literature suggesting that EMT and senescence are intrinsically linked, the causation or the correlation between these two processes has not yet been fully elucidated [15]. In addition, how SAS factors are regulated to either lead to tumour regression or promote tumour metastasis is not well understood. Thus, additional investigations of key senescence and EMT regulators are needed to define the identified phenotype-switching mechanism. Our results must be validated in different CRC in vitro/vivo or tumour-derived models. Finally, identifying the mechanistic pathways used by resistant cells (innately or acquired) under treatment pressure, if confirmed in clinical series, provides potential clues for future combinatory therapeutic strategies.

## 5. Conclusions

This study revealed the presence of senescence-like properties in five CRC cell lines short-term exposed to regorafenib leading to the investigation of a drug-initiated senescence-like phenotype as a cell death escape mechanism. More specifically, under prolonged exposure to regorafenib, SW480 cells demonstrated a stable therapy-induced phenotype (TIS), with most of these being arrested in the late G2/prophase cell cycle stage. In contrast, HCT-116 treated cells presented early senescent features and developed an acquired resistance triggering epithelial-mesenchymal transition (EMT) and a more aggressive phenotype over time. These data open new avenues for investigating major determinants of regorafenib-induced phenotype switching in CRC to further comprehend the poor efficacy of this drug in clinic.

## Data Availability

Not applicable.

## Supplementary Materials

The following supporting information can be downloaded at https://www.mdpi.com/article/10.3390/cells11223663/s1, Table S1: Colorectal Cancer (CRC) cell lines characteristics and experiment-related sending densities. MSI, microsatellite instability; Table S2: STR (Short Tandem Repeat) profiles of the original cell lines (SW480 and HCT-116 control group) and long-term treated cell lines (Rego-12m group); Table S3: List of qPCR primers with the relative forward and reverse sequences. All from Thermo Fisher Scientific, Waltham, MA, USA; Figure S1: Regorafenib effect cell proliferation and migration ability in SW480 cell line.
