# Supplementary material for: Regorafenib Induces Senescence and Epithelial-Mesenchymal Transition in Colorectal Cancer to Promote Drug Resistance
Source: Cells. 2022 Nov 18;11(22):3663. doi: 10.3390/cells11223663 (PMC9688587; doi:10.3390/cells11223663)
Supplement: Supplementary file 1 [file cells-11-03663-s001.zip › cells-2012438-supplementary.pdf]

Supplementary Table S1: Colorectal Cancer (CRC) cell lines characteristics and experiment-related sending densities. MSI, microsatellite instability.

Human CRC Cell line	Origin	Disease	Mutational Profile					MSI Status	Cell Viability Assay	Detection and Measurement of β-galactosidase Activity		Cell Cycle Analysis
			KRAS	PIK3CA	TP53	APC	*β-Catenin*		96 well plate, cells/well	4-chamber slides, cell/chamber	6 well plate, cells/well	
HCT-116	Large intestine, Colon	Colorectal Adenocarcinoma	x	x			x	Yes	$1,5\times10^{4}$	6×10^{3}	5×10^{5}	5×10^{5}
SW1116	Large intestine, Colon	Colorectal Adenocarcinoma	x		x	x		No	12×10^{3}	50×10^{3}	10×10^{5}	10×10^{5}
LS1034	Large intestine, Cecum	Colorectal Adenocarcinoma	x		x	x		No	4×10^{3}	15×10^{3}	5×10^{5}	5×10^{5}
SW480	Large intestine, Colon	Colorectal Adenocarcinoma	x		x	x		No	$5x10^{3}$	20×10^{3}	5×10^{5}	5×10^{5}
Caco-2	Large intestine, Colon	Colorectal Adenocarcinoma			x	x	x	No	6×10^{3}	20×10^{3}	5×10^{5}	5×10^{5}

Supplementary Table S2: STR (Short Tandem Repeat) profiles of the original cell lines (SW480 and HCT-116 control group) and long-term treated cell lines (Rego-12m group).

	Locus	Original cell line	Rego-12m		Locus	Original cell line	Rego-12m
**SW480**	*Amelanogenin*	X,X	X,X	**HCT-116**	*Amelanogenir*	X, Y	X,Y
	CSF1PO	13,14	13,14		CSF1PO	7,10	7,10
	D13S317	12	12,12		D13S317	10,12	10,12,13
	D16S539	13	13,13		D16S539	11,13	11,13
	D5S818	13	13,13		D5S818	10,11	10,11
	D7S820	8	8,8		D7S820	11,12	11,12
	THO1	8	8,8		THO1	8,9	8,9
	TPOX	11	11,11		TPOX	8,9	8,9
	vWA	16	16,16		vWA	17,22	16,17,18,22

Supplementary Table S3: List of qPCR primers with the relative forward and reverse sequences. All from Thermo Fisher Scientific, Waltham, MA, USA.

Gene	Forward Primer Sequence (5' → 3')	Reverse Primer Sequence (5' → 3')
**p16**	GATCCAGGTGGGTAGAAGGTC	CCCCTGCAAACTTCGTCCT
**p19**	AGTCCAGTCCATGACGCAG	ATCAGGCACGTTGACATCAGC
**p27**	ACGGGAGCCCTAGCCTGGAGC	TGCCCTTCTCCACCTCTTGCC
**TGF-β1**	TGAACCCGTGTTGCTCTCCCG	CTGCCGCACAACTCCGGTGA
**ZEB1**	AAGAATTCACAGTGGAGAGAAGCCA	CGTTTCTTGCAGTTTGGGCATT
**MMP9**	TGTACCGCTATGGTTACACTCG	GGCAGGGACAGTTGCTTCT
**N-Cad**	TCAGGCGTCTGTAGAGGCTT	ATGCACATCCTTCGATAAGACTG
**18S**	GCGGCGGAAAATAGCCTTTG	GATCACACGTTCCACCTCATC

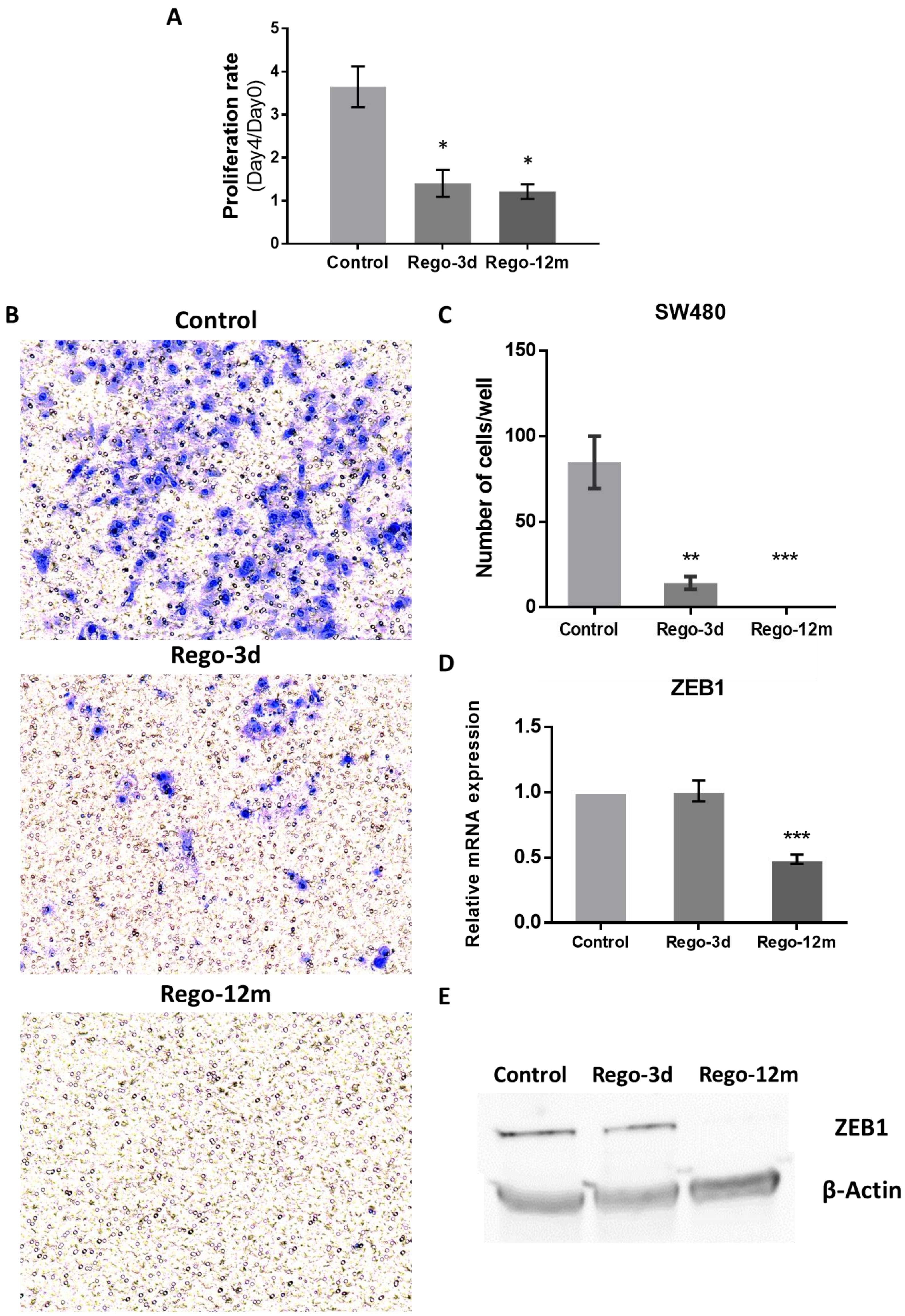

Supplementary Figure 1: Regorafenib effect cell proliferation and migration ability in SW480 cell line. **(A)** Proliferation rates of SW480 cells assessed by crystal violet**, (B)** Transwell assays were used to detect the migration capacity of cells. Representative pictures showing migration of cells in Control; Rego-3d (short regorafenib exposure (3 days)) and Rego-12m group (long regorafenib exposure (12 months)) (crystal violet staining, 10x magnification). **(C)** Column diagram indicating the amounts of migrating cells per field. **(D)** Relative gene expression level of *ZEB*1 determined by real-time PCR. **(E)** Protein expression level of ZEB1 assessed by Western Blot. β-Actin is used as loading control. Data are expressed as means ± SEM (n≥3) compared to control group with *P<0.05, **P<0.01, ***P<0.001 values from Student's t-test.
